# Exploring the Possible Impact of Oral Nutritional Supplements on Children’s Oral Health: An In Vitro Investigation

**DOI:** 10.3390/dj12030078

**Published:** 2024-03-19

**Authors:** Cynthia Anticona, Lena Hansson, Ingegerd Johansson, Pernilla Lif Holgerson

**Affiliations:** 1 Department of Odontology, Faculty of Medicine, Umeå University, 90187 Umeå, Sweden; 2 Department of Clinical Sciences, Pediatrics, Faculty of Medicine, Umeå University, 90187 Umeå, Sweden; lena.hansson@umu.se; 3 Department of Odontology, Section of Cariology, Faculty of Medicine, Umeå University, 90187 Umeå, Sweden; ingegerd.johansson@umu.se; 4 Department of Odontology, Section of Pediatric Dentistry, Faculty of Medicine, Umeå University, 90187 Umeå, Sweden; pernilla.lif@umu.se

**Keywords:** cariogenic potential, dental erosive potential, oral health, pediatric oral nutritional supplements

## Abstract

Eight pediatric oral nutritional supplements (ONSs) and 0.5% fat bovine milk were examined in vitro regarding their effect on the adhesion of three caries-related bacteria, *Streptococcus mutans* (strain CCUG 11877T), *Lactobacillus gasseri* (strain CCUG 31451), and *Scardovia wiggsiae* (strain CCUG 58090)*,* to saliva-coated hydroxyapatite, as well as their pH and capacity to withstand pH changes. Bacteria were cultivated and radiolabeled. The adhesion assays used synthetic hydroxyapatite coated with whole or parotid saliva. Measurements of pH and titration of the products with HCl and NaOH were conducted in triplicate. Three ONSs promoted the *S. mutans* adhesion to saliva-coated hydroxyapatite (increase from 35% to >200%), supporting caries risk enhancement. *S. wigssiae* and *L. gasseri* adhered only to one and no ONS, respectively. Most supplements had limited buffering capacity to counteract acidification changes, suggesting their low capacity to neutralize acids, and one ONS showed a significant capacity to counteract basic changes, suggesting a high erosive potential. *S. mutans* adhesion was influenced by the ONS pH and volume NaOH added to reach pH 10. *L. gasseri* and *S. wiggsiae* adhesion was influenced by the ONSs’ carbohydrate and fat content. Interdisciplinary efforts are needed to increase awareness and prevent the possible negative impact of ONSs on children’s oral health.

## 1. Introduction

Pediatric oral nutritional supplements (ONSs) are essential for the development and health of children with specific medical conditions. However, their composition may also pose a potential risk to oral health. ONSs are food products with a formulation of nutrients adapted to persons with specific diseases or conditions, and who are temporarily or permanently unable to meet nutritional requirements through oral diet alone [1]. In children, ONSs are used in the management of faltering growth and malnutrition associated with various diseases (e.g., cancer, cardiac disease) or underlying conditions (e.g., feeding difficulties, endocrine disorders, neurodevelopmental disorders, genetic syndromes, and prematurity) [1]. Positive effects of ONSs on weight gain, height growth, nutritional intake, and reduction in infections have been reported in children with or at risk of faltering growth, associated or not to feeding disorders [2,3,4,5,6]. Pediatric feeding disorders have been reported to affect 9 to 25% of children in the general population and 80% of children with developmental disabilities [7,8]. This prevalence is, however, expected to increase due to the improved survival of severely ill or developmentally disabled children [9], which also implicates an increased market of ONS [10].

ONS are available in several formats and textures (ready-to-drink, dessert, juice), with different fiber contents, volumes, and energy densities, and are prescribed based on age and medical condition, either as a dietary supplement or the sole source of nutrition [1]. To meet the recommended nutritional needs for children, ONSs contain macronutrients and micronutrients [11], with carbohydrates also providing texture and taste to improve compliance [12]. The carbohydrates in ONSs include starch-derived polymers, i.e., maltodextrins and glucose syrups, which are widely used in nutritional products to increase the caloric content and provide a thickening effect [13], and also sucrose, which makes ONSs highly cariogenic [14].

Dental caries is one of the most prevalent chronic diseases worldwide, though with large variations between and within countries [15]. The disease results from the action of acidic metabolites from carbohydrate fermentation, such as the carbohydrates in ONSs, through bacteria in the tooth-colonizing communities [16]. Hence, the abundance of aciduric and acid-tolerant species, such as *Streptococcus mutans*, *Scardovia wiggsiae,* and lactobacilli, in the tooth biofilm, and especially in dysbiosis, is a well-known risk factor for childhood caries. The abundance of such species is basically driven by their ability to adhere to saliva epitopes or partner bacteria, and to thrive at low pH [17,18,19]. In addition to caries, an acidic environment may lead to wear of the teeth (dental erosion) [20].

Accordingly, there is a strong association between caries and the consumption of free sugars, i.e., mono- and disaccharides, in drinks and foods, and sugars naturally present in honey, syrups, fruit juices, and concentrates [21]. General guidelines recommend a maximum intake of free sugars of 10% of the total energy intake per day (E%) [18], with European recommendations having a lower limit for children aged 2–18 years (<5 E%), and even lower for infants [22]. Similarly, North American guidelines for children 2 years and older specify a consumption of added sugars ≤ 25 g per day and a maximum 8 oz of sugary drinks per week [23]. However, an exception is noted for individuals in need of therapeutic diets, for whom the high sugar intakes can be justified for medical reasons [21]. Although medically justified, children consuming ONSs have a high sugar intake and subsequent increased caries risk, which likely worsens if the child’s physical limitations and/or disease management require frequent feeding or constrain oral hygiene habits. Similarly, the presence of associated diseases/conditions and nutritional deficits can disturb the development of dental tissues and the defense mechanisms of saliva, with a subsequent increased caries risk [24].

This in vitro study was motivated by the need to investigate whether pediatric ONSs can harm oral health and call for the introduction of a targeted prevention program. Eight ONSs and 0.5% fat bovine milk were selected for an examination of (i) their effect on adhesion of *S. mutans*, *Lactobacillus gasseri*, and *S. wiggsiae* to saliva-coated hydroxyapatite, and (ii) their pH and capacity to withstand pH changes.

## 2. Materials and Methods

### 2.1. Test Products

Eight ready-to-drink ONSs specifically developed for the treatment of pediatric malnutrition and frequently prescribed in Sweden were evaluated in the present study. Bovine milk was used as a reference and was also positive for *S. mutans* control since it is known to have a significant inhibitory effect on *S. mutans* and a buffering capacity [25,26].

The ONS products varied in terms of the (i) target age, (ii) format, (iii) volume and taste, (iv) nutritional content, and (v) producer (Table 1): ONS 1 (Fortini Multi Fibre^®^, Nutricia, Utrecht, The Netherlands/200 mL), ONS 2 (Fortini^®^, Nutricia, Utrecht, The Netherlands/200 mL), ONS 3 (Fortini Smoothie^®^, Nutricia, Utrecht, The Netherlands/200 mL), ONS 4 (Fortini Compact Multi Fibre^®^, Nutricia, Utrecht, The Netherlands/125 mL), ONS 5 (Frebini energy fibre drink^®^, Fresenius- Kabi, Bad Homburg v.d.H, Germany/200 mL), ONS 6 (Frebini energy drink^®^, Fresenius- Kabi, Bad Homburg v.d.H, Germany/200 mL), ONS 7 (Resource MiniMax^®^, Nestle, Vevey, Switzerland/200 mL), and ONS 8 (Infatrini^®^, Nutricia, Utrecht, The Netherlands/200 mL). ONSs indicated for the management of a specific disease (e.g., diabetes) and products designed for administration via a percutaneous endoscopic gastrostomy tube were not included. Most of the tested products are intended for children > 1 year, and only one (ONS 8) is recommended for infants < 18 months. ONS 4 is a 125 mL drink with a higher nutrient density to equate to the composition of the typical 200 mL drinks. In contrast, ONS 8 with lower nutrient density aims to meet the nutritional requirements in infants. Six of the tested ONSs have a carbohydrate content between 14 and 19 g/100 mL, whereas one (ONS 4) contains 29 g/100 mL and ONS 8 contains 10 g/100 mL. In comparison, 0.5% fat bovine milk only contains 5% carbohydrate (lactose). Apart from ONS 4 and 8, the protein and fat contents were similar in all test products but only half of them (n = 4) contained fiber (Table 1).

### 2.2. Saliva Collection

This study used anonymized samples of whole and parotid saliva collected in a previous study and stored at −80 °C. Whole saliva from healthy children aged 0–5 years was collected for up to two minutes using a suction device attached to slight vacuum from the dental unit, according to the procedure described elsewhere [27], and then pooled. Parotid saliva from one healthy adult was collected using Lashley cups placed over the parotid orifices and during saliva flow stimulation, with acid taste as described elsewhere [28]. The tubes into which whole and parotid saliva was collected were stored on ice during the collection and then transferred to a −80 °C freezer where they were stored until being retrieved for use.

### 2.3. Bacteria Cultivation and Radiolabeling

S. *mutans* (strain CCUG 11877T), *L. gasseri* (strain CCUG 31451), and *S. wiggsiae* (strain CCUG 58090) were transferred from frozen stocks in defatted milk to blood (S. *mutans* and *S. wiggsiae*) or Rogosa agar (*L. gasseri*) plates and grown for up to 72 h at 37° in 5% CO_2_ (*S. mutans* and *S. wiggsiae*) or anaerobically (*L. gasseri*). Single colonies of each species were picked and recultivated for 24 h under the same conditions. Grown bacteria were harvested using a cotton swab and suspended in an 80 µL M-dil solution (4.4 g NaCl, 0.42 g KCl, 1.0 g Na_2_HPO × 2H_2_O, 1.0 g KH_2_PO_4_, 10.0 g C_3_H_9_Na_2_O_7_P × H_2_O mixed with 0.1 g MgC_l2_ × 6H_2_O, 500 mL distilled water), together with 400 µCi (20 µL EasyTag EXPRESS 35S Protein Labeling Mix, PerkinElmer, Hägersten, Sweden), and spread on pre-warmed blood agar plates (*S. mutans* and *S. wiggsiae*) or Rogosa agar (*L. gasseri*) and grown overnight as described above. The bacteria were harvested after 24 h using a cotton swab and suspended in ADH buffer (ADH; 50 mM KCl, 1 mM CaCl_2_•2H_2_O, 0.1 mM MgCl_2_•6H_2_O, 0.62 mM K_2_HPO_4_, 1.4 mM KH_2_PO_4_, pH 6.5) with 5% bovine serum albumin (BSA, Sigma Aldrich Inc., St. Louis, MO, USA) to an optical density corresponding to 1 × 109 cells/mL.

### 2.4. Bacterial Adhesion Assay

Bacterial adhesion to saliva-coated hydroxyapatite was assessed as described by Gibbons and Hay [29]. Briefly, 5 mg of synthetic hydroxyapatite (HA) beads (Macro-Prep Ceramic Hydroxyapatite Type II, BioRad, CA, USA) were placed in each well of a 96-well round-bottom microtiter plate (Nunclon Surface, NUNC, Roskilde, Denmark). After overnight equilibration in 125 µL ADH at 4 °C, the beads were washed once with ADH buffer, and incubated with 125 µL pooled child saliva or 125 µL parotid saliva (both diluted 1:1) for 1 h at room temperature under slow rotation. After three washes with ADH buffer, the beads were treated with 5% bovine serum albumin (BSA, Sigma Aldrich Inc., St. Louis, MO, USA) in water for one hour at room temperature to block any uncoated parts of the HA beads. After three washes with ADH, 62.5 µL of the eight ONS (undiluted), undiluted 0.5% fat bovine milk, or ADH, respectively, were mixed with 62.5 µL radiolabeled bacteria suspension, and incubated under constant rotation for 1 h at room temperature. The number of bacteria attached, after unbound bacteria were washed away, was determined by scintillation counting (MicroBeta 2, PerkinElmer, Hägersten, Sweden), and the proportion of bound bacteria out of the total amount of added bacteria (percent adhesion) was calculated. All measures were performed in triplicate.

### 2.5. pH Measurement and Titration of the ONS

The pH of the eight ONS and 0.5% fat bovine milk was measured in 20 mL undiluted samples using a Mettler Toledo Seven Easy S20 (Thermo Fisher Scientific, Greifensee, Switzerland) pH meter. Before use, the pH electrode was calibrated using pH 4 and pH 7 standard buffers, and between measurements, the electrode was carefully rinsed with Milli-Q ultrapure water (Merck Life Science AB, Solna, Sweden). During the measurements, the samples were stirred with a non-heating magnetic stirrer until the pH was stabilized. The pH values of 0.5% fat bovine milk and seven ONSs with initial pH > 6 were measured after stepwise additions of 1 mL aliquots of 0.1 M HCl (strong acid) and of 0.5% fat bovine milk and ONS 3 with initial pH < 4 after the addition of 1 mL aliquots of 1 M NaOH (strong base). Furthermore, the amounts of 0.1 M HCl needed to reach a pH of 5 for 0.5% fat bovine milk and the ONSs with initial pH > 6 and the amount of 1 M NaOH to reach pH 10 for 0.5% fat bovine milk and ONS 3 were recorded. All measurements were performed in triplicate.

### 2.6. Analysis

The mean and standard deviation (SD) of triplicate measurements of (i) bound bacteria to pooled child saliva (s-HA) or adult parotid saliva (ps-HA) and (ii) pH of the eight ONS and 0.5% fat bovine milk were calculated. The capacity to inhibit or promote bacterial adhesion was tested by calculating the percentage difference between the mean adhesion to s-HA alone and after the addition of each of the test products. Partial least square regression (PLS) modelling was performed to examine the correlation structure between the bacterial adhesion of each of the three species (dependent variables) with potential explanatory variables (ONS composition, pH, and adhesion pattern of the two other bacteria not occupied as dependent variable). The descriptive analyses were performed using Stata (version 14, StataCorp LLC, College Station, TX, USA) and PLS analysis using SIMCA (version 18, Sartorius Stedim Data Analytics AB, Umeå, Sweden).

## 3. Results

### 3.1. ONS Mediation of the Adhesion of S. mutans, L. gasseri, and S. wiggsiae to s-HA

**Figure 1:**
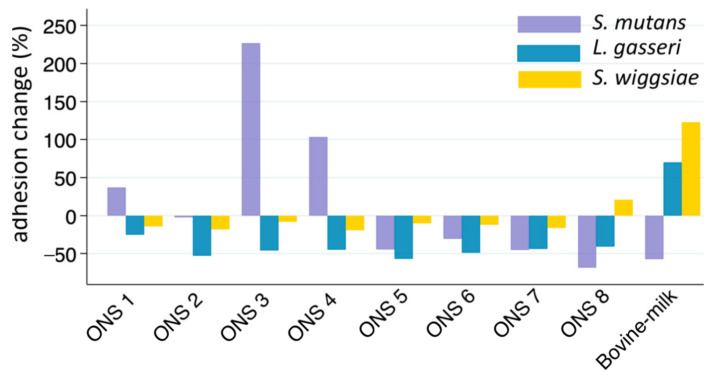
Bacterial adhesion change (%) following the addition of eight oral nutritional supplements (ONSs) and 0.5% fat bovine milk to saliva-coated hydroxyapatite. Negative values indicate binding inhibition and positive values increased binding.

The adhesion of *S. mutans*, *L. gasseri*, and *S. wiggsiae* to s-HA and ps-HA, and the impact of adding each of the eight test products in the s-HA assay, are presented in Table 2 and Figure 1. The binding of bacteria to HA coated with children’s whole saliva was generally lower than that observed with HA coated with adult parotid saliva. This difference was particularly noticeable for *S. mutans*, but it was also observed for *L. gasseri* and *S. wiggsiae*, as shown in Table 2. The introduction of ONS products either increased or decreased the binding to s-HA for all ONS, except for ONS 2. The most notable change was observed for *S. mutans*, where binding increased from 5.1% to 16.5% with the addition of ONS 3. The most effective inhibition of *S. mutans* binding was with ONS 8, where binding dropped from 5.1% to 1.6% (Table 2). Consequently, the attachment of *S. mutans* to s-HA increased by over 200%, 100%, and 35% with the addition of ONSs 3, 4, and 1, respectively, compared to its binding to HA only coated with children’s saliva (Figure 1). Conversely, the binding decreased for the other ONSs and 0.5% fat bovine milk, with a notable decrease of 68% for ONS 8, which was even more pronounced than the decrease caused by 0.5% fat bovine milk (49%).

The adhesion of *S. wiggsiae* to s-HA increased by 123%, with the addition of 0.5% fat bovine milk, and also rose moderately, by 21.5%, with the addition of ONS 8. However, it decreased following the introduction of all the other ONS products. Similarly, the adhesion of *L. gasseri* to s-HA enhanced somewhat after the addition of 0.5% fat bovine milk, increasing from 1.9% to 3.2%, but was inhibited by the eight tested ONS products (Table 2 and Figure 1).

### 3.2. pH and Buffering Capacity

The baseline pH of eight test products and 0.5% fat bovine milk was assessed, along with their resistance to pH changes after the addition of a strong acid (HCl) or base (NaOH). The volumes of 0.1 M HCl needed to reduce the pH to 5 (critical pH for caries demineralization) for products with a baseline pH above 6.3, or of 1 M NaOH to increase the pH to 10 for one product with an acidic baseline pH of 3.5 (ONS 3, i.e., with a tooth tissue erosive potential) were measured. The amount of HCl needed to decrease pH to 5 varied from 3.0 to 7.5 mL, with ONSs 1 and 8 requiring the least acid and ONS 5 the most (Table 3). ONS 3 needed 7 mL 1 M NaOH to raise the pH from 3.5 to 10 (Table 3).

**Figure 2:**
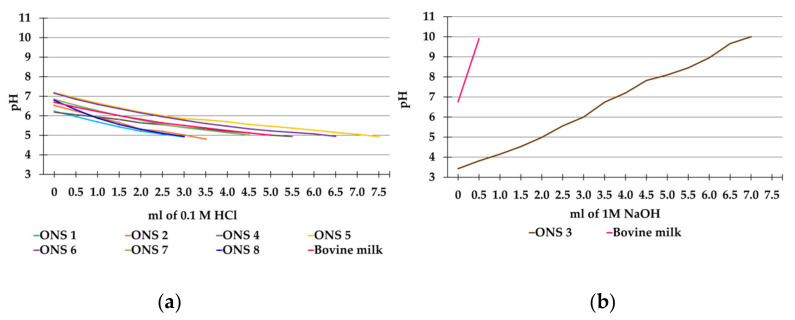
Line plots of pH changes following the addition of a strong acid (HCl) or base (NaOH). (**a**) pH changes when adding 0.1 M HCl to seven oral nutritional supplements (ONSs) and 0.5% fat bovine milk to reduce the pH to 5 (**b**) pH changes when adding 1 M NaOH to ONS 3 and 0.5% fat bovine milk to raise the pH to 10.

Additionally, the pH changes were followed during titration with 0.1 M HCl from the neutral baseline pH to around 5 for products with a near-neutral baseline pH. All these products followed a parallel linear decrease with increasing acid addition but with a clear distinction in the slope of the curves (Figure 2a). Thus, ONSs 1 and 8 had the steepest slopes, ONS 5 the least-steep slope, and that of 0.5% fat bovine milk was intermediary (Figure 2a). For the acidic ONS 3 and 0.5% fat bovine milk, the pH changes were monitored while titrating with 1 M NaOH. The 0.5% fat bovine milk showed a rapid pH increase upon the addition of NaOH, requiring about 0.6 mL to reach pH 10, whereas the pH of ONS 3 increased continuously while more than 10 times as much base. A total of 3 mL of 1M NaOH was needed to reach a pH of 6, and 7 mL to reach pH 10, which is over seven times more than what was needed for 0.5% fat bovine milk to reach the same pH (Figure 2b).

### 3.3. Multivariate Characterization of Bacterial Adhesion versus ONS Compositions

To search for factors associated with the change in bacterial adhesion when the ONS products were added to the s-HA test assay, multivariate PLS models with adhesion changes for each of the test strains as the dependent variables were run. The independent variables included the main components in each ONS and 0.5% fat bovine milk, pH-related factors, and adhesion to s-HA of the two complementary bacteria. All three models were strong, with explanatory powers (R^2^) of the two first components exceeding 87% and predictive powers exceeding 43% (Table 4). The pH values of the ONS and the volume of NaOH added to reach pH 10 were the most influential factors for the variation in *S. mutans* adhesion, with a variable importance in projection value (VIP) > 1.5. The effect on *L. gasseri* adhesion was paralleled by *S. wiggsiae* binding to s-HA (VIP = 1.8) and influenced by the carbohydrate and fat content in the ONS products (VIP 1.2 and 1.4, respectively). In line with this, the effect on *S. wiggsiae* adhesion was associated with *L. gasseri* binding to s-HA as well as the carbohydrate and fat content of the ONS (VIP = 1.3 and 1.5, respectively).

## 4. Discussion

This in vitro study examined two key aspects in relation to their potential for dental harm. Firstly, it assessed the impact of ONSs on the initial adherence to the tooth-mimicking HA of three bacteria implicated in caries disease and especially in early childhood caries. Secondly, the study evaluated the capability of ONSs with a neutral pH to maintain it at a non-cariogenic level under acidic exposure. Additionally, for one acidic ONS formula, its behavior was tested when exposed to a base as a measure of its erosive potential. The key findings were that the ONSs tested had a significant impact on *S. mutans* adhesion to s-HA, and that most ONSs conferred limited buffering to counteract the acidification from HCl, suggesting that in the oral cavity these products would have a low capacity to neutralize acids produced by cariogenic bacteria or introduced through other foods/drinks. Furthermore, the acidic ONS formula showed a large buffering capacity to counteract the addition of NaOH, suggesting a high dental erosive potential.

The composition of tooth colonizing bacterial communities (dental plaque) is one cornerstone in a person´s risk of developing caries. The ecology of dental plaque is complex, with influences from host factors, neighboring bacteria, and external components, e.g., foods. It is organized in spatial consortia, for which adhesion to saliva is one important determinant. Generally, bacterial biofilms are protective, but a dysbiotic biofilm may evolve with the adhesion of disease-associated species of aciduric and acidogenic properties such as *S. mutans, S. wiggiae,* and *L. gasseri.* The adhesion of *S. mutans* to s-HA was promoted by three ONS formulas tested in this study, with the largest effect shown for the acidic formula ONS 3 and inhibited by the remaining five, with the largest inhibitory effect seen for ONS 8. Previous studies have shown that bovine milk inhibits *S. mutans* attachment to ps-HA [30,31], whereas the effect of human milk addition varies by milk and saliva donor [28]. Thus, the milk inhibition of *S. mutans* binding here is in line with published data, but other results cannot be compared since this is the first study to evaluate the effect of milk-based nutritional supplements.

Notably, the PLS regression results indicated that acidic pH levels and a high buffer capacity in ONSs are linked to the increased adhesion of *S. mutans* to s-HA. This supports the existing understanding that *S. mutans* prospers in acidic environments [17]. While individual ONS products did affect *S. mutans* binding, their major nutritional components did not seem to influence this adhesion. However, we cannot discount the possibility that the compositional differences among the few tested products were too narrow to detect effects.

The suppressive effect of bovine milk on *S. mutans* adherence to HA is credited to certain proteins, such as caseins and lactoferrin [30,31], and also to unsaturated fatty acids [25]. Most ONS formulas examined in this study, except ONS 3, contained casein from bovine milk as a protein source. Nevertheless, the potential caries-preventive role of bovine milk components might be offset by the addition of non-milk carbohydrates, especially sucrose [32]. This is supported by prior studies demonstrating the cariogenicity of sugar-rich, milk-based nutritional supplements for elderly individuals [32,33,34], and by findings that the casein content did not reduce the cariogenicity of commercial infant formulas containing extrinsic sugars (e.g., sucrose, glucose) [35].

Compared to *S. mutans*, the influence of 0.5% fat bovine milk and the tested oral nutritional supplements on the adhesion of *L. gasseri* and *S. wiggsiae* to s-HA was less pronounced, and their patterns were contrasting. Here, 0.5% fat bovine milk enhanced the binding of both *L. gasseri* and *S. wiggsiae*, while all ONSs, except for ONS 8 for *S. wiggsiae*, reduced binding. Moreover, the PLS analyses revealed that the adhesion of *L. gasseri* and *S. wiggsiae* to s-HA paralleled each other, and the inhibition of adhesion for both was linked to the carbohydrate and fat content in the ONS. This observation suggests a potential similarity in at least one binding epitope between the two species, and possibly carbohydrate-related epitopes, as, besides the carbohydrate content per se, many of the fats in milk are glycolipids [36].

Out of the eight tested ONSs, only ONS 3 had an acidic pH, likely due to its content of fruit juice/puree. Thus, the low pH of ONS 3, combined with its capacity to maintain a low pH even at substantial amounts of base addition, gives ONS 3 a significant dental erosive potential. Fruit-juice-based ONSs are still rare compared to regular milk-based ONSs [34]; however, the range is increasing due to positive effects on compliance, especially in individuals with an altered sensory sensitivity, poor appetite, or avoidance of certain foods. Their use is also considered a good strategy to avoid taste fatigue and monotony, which tend to occur when ONSs are consumed regularly over prolonged periods [6]. Despite these benefits, ONSs containing fruit juice have elevated contents of sugar and acids [37]. Hence, their cariogenic and erosive potential should be acknowledged, and their use should be accompanied by collaborative prevention strategies at individual level, and especially in young children with sensitive deciduous or newly erupted permanent teeth.

This study highlights that the use of ONSs, while nutritionally urged, may also carry a risk for tooth harm due to their content of carbohydrates that are readily fermented by oral bacteria. However, there are limitations in the study that should be considered before drawing definitive clinical conclusions. Firstly, the limited number of ONS products analyzed restricted our ability to thoroughly assess changes in bacterial adhesion patterns following ONS exposure and the underlying factors of these changes. Secondly, the evaluation of ONS’s cariogenic effect would have been more definitive if a standardized cariogenicity assessment method had been utilized. Such methods might include measuring sugar fermentability, plaque acidification, animal testing, and demineralization–remineralization models, or conducting epidemiological studies in children [38]. Despite these limitations, the influence of carbohydrates in ONSs on bacterial acid production and the development of caries is supported by numerous studies on sugars and processed starches [14]. In our research, we focused on additional aspects, examining the impact of ONSs on the adhesion of bacteria associated with caries and the buffering effects of the ONS. We suggest that future studies evaluate ONS effects by one or more of the mentioned methods, using multispecies biofilms, and evaluate the impact of the viscosity of the ONS. Viscosity was not considered in the present study, but the proportionally high contents of simple sugars, maltodextrins, and glucose syrups imply that ONSs are viscous with prolonged clearance times [39,40].

## 5. Conclusions

The conclusions can be summarized as follows:

- Some of the ONSs examined in this in vitro study promoted the adhesion of *S. mutans* to synthetic HA, which supports their cariogenic potential.
- One ONS was highly acidogenic, suggesting an erosive dental potential as well.
- Collaborative interdisciplinary efforts, particularly between oral health and nutrition professionals, are needed to both increase awareness of the possible negative impact of these supplements for oral health and to provide reinforced preventive oral care following the prescription.

## Figures and Tables

**Table 1:** General characteristics of the eight studied ready-to-drink oral nutritional supplements (ONS) and bovine milk.

Product	Target Age	Formulation, Volume, and Taste	Kcal per 100 g/mL	Carbohydrates per 100 g/mL	Proteins per 100 g/mL	Fat per 100 g/mL	Fiber	Producer
ONS 1: Fortini Multi Fibre	1–12 years	200 mL, chocolate	153 kcal	18.8 g	3.3 g	6.8 g	Yes	Nutricia, Utrecht, The Netherlands
ONS 2: Fortini	1–12 years	200 mL, vanilla	150 kcal	18.8 g	3.3 g	6.8 g	No	Nutricia, Utrecht, The Netherlands
ONS 3: Fortini Smoothie	1–12 years	200 mL, summer fruits	150 kcal	19.0 g	3.4 g	6.4 g	Yes	Nutricia, Utrecht, The Netherlands
ONS 4: Fortini compact multifiber	1–12 years	125 mL, neutral	240 kcal	28.5 g	5.7 g	10.9 g	No	Nutricia, Utrecht, The Netherlands
ONS 5: Frebini energy fibre drink	1–12 years	200 mL, chocolate	150 kcal	18.1 g	3.8 g	6.7 g	Yes	Fresenius- Kabi, Bad Homburg v.d.H, Germany
ONS 6: Frebini energy drink	1–12 years	200 mL, strawberry	150 kcal	18.7 g	3.8 g	6.7 g	No	Fresenius- Kabi, Bad Homburg v.d.H, Germany
ONS 7: Resource MiniMax	1–15 years	200 mL, chocolate	121 kcal	13.7 g	3.9 g	5.6 g	No	Nestlé, Vevey, Switzerland
ONS 8: Infatrini	0–18 months	200 mL, neutral	100 kcal	10.2 g	2.6 g	5.3 g	Yes	Nutricia, Utrecht, The Netherlands
Bovine milk	-	-	40 kcal	5.0 g	3.4 g	0.5 g	No	-

**Table 2:** Mean (%) and standard deviation (SD) of triplicate measures of adhesion of *Streptococcus mutans*, *Lactobacillus gasseri*, and *Scardovia wiggsiae* to saliva-coated hydroxyapatite (child whole saliva and adult parotid saliva) and percentage difference (% diff) of adhesion after exposure to eight oral nutritional supplements (ONS) or 0.5% fat bovine milk.

	*S. mutans*		*L. gasseri*		*S. wiggsiae*	
	Mean (SD)	% diff	Mean (SD)	% diff	Mean (SD)	% diff
s-HA * + ADH	1.8 (0.3)		1.0 (0.1)		1.8 (0.5)	
ps-HA ** + ADH	12.9 (3.3)		1.3(0.1)		4.6 (0.5)	
s-HA	5.1 (0.9)		1.9 (0.3)		3.1 (0.3)	
s-HA + ONS 1	6.9 (1.7)	37.0	1.3 (0.2)	−25.0	2.8 (0.2)	−114.5
s-HA + ONS 2	5.0 (0.3)	−2.2	0.9 (0.2)	−52.9	2.5 (0.3)	−18.2
s-HA + ONS 3	16.5 (1.2)	226.7	1.0 (0.1)	−45.8	2.8 (0.8)	−8.3
s-HA + ONS 4	10.3 (1.6)	103.3	1.0 (0.0)	−44.8	2.5 (0.5)	−19.0
s-HA + ONS 5	2.9 (0.2)	−44.7	0.8 (0.2)	−56.7	2.7 (0.4)	−9.5
s-HA + ONS 6	3.3 (0.7)	−30.3	0.9 (0.2)	−49.4	2.8 (0.2)	−12.0
s-HA + ONS 7	2.8 (0.3)	−45.3	1.0 (0.2)	−43.5	2.6 (0.1)	−15.7
s-HA + ONS 8	1.6 (0.1)	−68.6	1.0 (0.2)	−40.6	3.7 (0.2)	21.5
s-HA + 0.5% fat bovine milk	2.2 (0.4)	−57.4	3.2 (0.1)	70.0	6.8 (0.8)	123.1

* HA coated with pooled child whole saliva. ** HA coated with parotid saliva from one adult person.

**Table 3:** Mean with standard deviation (SD) of triplicate measures of baseline pH of eight oral nutritional supplements (ONSs) and 0.5% fat bovine milk and volume of 0.1 M HCl and 1 M NaOH to reduce the pH to 5 or raise it to 10, respectively.

Product	Initial pH Mean (SD)	mL HCl to Reach pH 5	mL NaOH to Reach pH 10
ONS 1	6.39 (0.01)	3.0	NA
ONS 2	6.67 (0.00)	3.5	NA
ONS 3	3.51 (0.01)	NA	7.0
ONS 4	6.39 (0.02)	5.5	NA
ONS 5	7.21 (0.03)	7.50	NA
ONS 6	7.20 (0.03)	6.50	NA
ONS 7	6.86 (0.02)	4.5	NA
ONS 8	6.85 (0.02)	3.0	NA
0.5% fat bovine milk	6.81 (0.02)	5.0	0.6

**Table 4:** Partial least square regression (PLS) model strength and variable importance in projection (VIP) for the main components in the oral nutritional supplements (ONSs) and 0.5% fat bovine milk, pH-related factors, and adhesion of the two complementary bacteria from multivariate PLS models with differences in bacterial adhesion before and after exposure to the respective ONS as dependent variables.

	*S. mutans* R^2^ = 0.96, Q^2^ = 0.76		*L. gasseri* R^2^ = 0.87, Q^2^ = 0.43		*S. wiggsiae* R^2^ = 0.91, Q^2^ = 0.54	
	VIP Value	Direction	VIP Value	Direction	VIP Value	Direction
Nutritional characteristics						
Carbohydrate content	1.02	+	1.19	+	1.33	+
Protein content	0.54	+	0.88	+	0.71	+
Fat content	0.88	+	1.38	+	1.45	+
Fiber	0.83	+	0.64	+	0.67	+
						
pH and pH changes						
Baseline pH	1.63	+	0.51	+	0.46	+
Volume of HCl to reach pH 5	0.20	+	0.18	+	0.27	+
Volume of NaOH to reach pH 10	1.56	+	0.41	+	0.44	+
						
Adhesion of other bacteria						
Adhesion of *S. mutans* to s-HA	NA	NA	0.85	+	0.77	+
Adhesion of *L. gasseri* to s-HA	0.72	+	NA	NA	1.76	+
Adhesion of *S. wiggsiae* to s-HA	0.77	+	1.81	+	NA	NA

The explanatory (R^2^) and predictive (Q^2^) values were 0.963 and 0.765 for the *S. mutans* model, respectively; 0.870 and 0.430 for the *L. gasseri* model, respectively; and 0.910 and 0.540 for the *S. wiggsiae* model, respectively.

## Data Availability

The datasets analyzed during this study are available from the corresponding author on reasonable request.
